# Preparation and Preservation of Ointments

**Published:** 1845-08

**Authors:** 


*Preparation and Preservation of Ointments:*—M. Deschamps, in "*The Journal de Pharmacie*," has thrown out a suggestion upon this subject which deserves attention. He made many experiments, he says, first, to ascertain whether the several varieties of fat may, in all cases, be used indiscriminately ; and, second, whether any means can be devised to prevent fats from becoming rancid, which must greatly impair their value. He found that an ointment, prepared by heating the buds of the *poplar* in melted lard, is subject to very little alteration by keeping ; and it therefore occurred to him that, as this may depend upon a portion of resin extracted from the poplar buds, a small proportion of gum benzoin might answer a similar purpose. On preparing these ointments and keeping them for several years, he found they had undergone no change, no approach to rancidity. Iodide of potassium is a very excellent test of any acidity in fat. And by this test he found that no admixture with fat tends to preserve it from change so well as benzoin or poplar buds ; the latter produces an orange-yellow colour, but its colour is not affected by long keeping, even mixed with acetate of lead.

Fat or lard, thus prepared with poplar buds, or gum bezoin, then, is the best possible basis for ointments containing metallic substances, red oxide of mercury, acetate of lead, iodide of potassium, &c. ; with essential oils it makes lip-salve, and an application to blisters very much preferable to ordinary ointments.—*Lond. Lan.*

